# Dengue nowcasting in Brazil by combining official surveillance data and Google Trends information

**DOI:** 10.1371/journal.pntd.0012501

**Published:** 2025-08-18

**Authors:** Yang Xiao, Guilherme Soares, Leonardo Bastos, Rafael Izbicki, Paula Moraga

**Affiliations:** 1 Computer, Electrical and Mathematical Sciences and Engineering Division, King Abdullah University of Science and Technology (KAUST), Thuwal, Saudi Arabia; 2 Institute of Mathematical and Computer Sciences, University of São Paulo (USP), São Carlos, Brazil; 3 Scientific Computing Programme, Oswaldo Cruz Foundation (Fiocruz), Rio de Janeiro, Brazil; 4 Department of Statistics, Federal University of São Carlos (UFSCar), São Carlos, Brazil; Beijing Children’s Hospital Capital Medical University, CHINA

## Abstract

Dengue is a mosquito-borne viral disease that poses significant public health challenges in tropical and sub-tropical regions worldwide. Surveillance systems are essential for dengue prevention and control. However, traditional systems often rely on delayed data, limiting their effectiveness. To address this, nowcasting methods are needed to estimate underreported cases, enabling more timely decision-making. This study evaluates the value of using Google Trends indices of dengue-related keywords to complement official dengue data for nowcasting dengue in Brazil, a country frequently affected by this disease. We compare various nowcasting approaches that incorporate autoregressive features from official dengue cases, Google Trends data, and a combination of both, using a naive approach as a baseline. The performance of these methods is evaluated by nowcasting weekly dengue cases from March 2024 to January 2025 across Brazilian states. Error measures and 50% and 95% coverage probabilities reveal that models incorporating Google Trends data enhance the accuracy of weekly nowcasts across states and offer valuable insights into dengue activity levels. To support real-time decision-making, we also present Dengue Tracker, a website that displays weekly dengue nowcasts and trends to inform both decision-makers and the public, improving situational awareness of dengue activity. In conclusion, the study demonstrates the value of digital data sources in enhancing dengue nowcasting, and emphasizes the value of integrating alternative data streams into traditional surveillance systems for better-informed decision-making.

## Author summary

Dengue is a mosquito-borne viral disease that poses significant public health challenges in tropical and sub-tropical regions worldwide. Surveillance systems are crucial for dengue prevention and control. Unfortunately, traditional systems often rely on delayed data, limiting their effectiveness. To address this, nowcasting methods are needed to estimate underreported cases, enabling more timely decision-making. This study evaluates how Google Trends indices of dengue-related keywords can complement official dengue data to improve nowcasting of dengue in Brazil, a country frequently affected by this disease. We compare the performance of various nowcasting approaches that incorporate Google Trends data with other approaches that rely solely on official reported cases data, assessing their accuracy and uncertainty in nowcasting weekly dengue cases from March 2024 to January 2025 across Brazilian states. To support real-time decision-making, we also present Dengue Tracker, a website that displays weekly dengue nowcasts offering valuable insights into dengue activity levels. The study demonstrates the potential of digital data sources in enhancing traditional surveillance systems for better-informed decision-making.

## Introduction

Dengue is a vector-borne disease transmitted by *Aedes* mosquitoes, which poses a significant global health threat, particularly in tropical and subtropical regions [1]. Dengue symptoms range from mild (fever, headache, rash, and muscle and joint pain) to severe forms like dengue hemorrhagic fever, which can cause bleeding, plasma leakage, and organ failure, increasing the risk of death [2]. While the disease does not spread from person to person, it can still lead to large outbreaks, especially in densely populated urban areas where mosquitoes breed in stagnant water. Several factors contribute to the prevalence of dengue, including climate, urbanization, and socio-economic disparities [3]. Additionally, global warming and increased international travel have expanded the geographic range of dengue [4].

In 2024, Brazil faced a severe dengue epidemic, with 9.48 million suspected cases and 5.32 million confirmed cases as of August [5]. This surge has made Brazil the most affected country in the Americas. The increased incidence is thought to be possibly attributed to factors like early transmission seasons, climate change, and the presence of all four dengue serotypes [6,7].

The extensive impact of dengue underscore the importance of research and the ability to predict its outbreaks. Surveillance systems play a crucial role for guiding strategies for prevention and control, but traditional surveillance systems often rely on delayed or incomplete data due to underreporting, healthcare infrastructure limitations, and time lags in laboratory testing. For example, in Brazil, a suspected dengue case is required by law to be reported by authorized health professionals in the Notifiable Diseases Information System (SINAN) [8]. However, according to [9], less than 50% of dengue cases are reported within the first week, no more than 75% are reported within four weeks, and fewer than 90% are reported within nine weeks. This latency necessitates nowcasting methods to estimate occurred-but-not-yet-reported disease cases for real-time decision-making.

Several nowcasting techniques have been developed in different settings. For example, [10] employed reverse-time discrete hazard functions and maximum likelihood estimation to handle reporting delays and nowcast AIDS cases in Canada. A Bayesian hierarchical model was proposed by [11] to improve prediction and management of a Shiga toxin producing Escherichia coli that caused a major outbreak in Germany in 2011. The model combined a survival regression model for the delay distribution, and a quadratic spline for the epidemic curve, utilizing the generalized Dirichlet distribution for flexibility in handling uncertainty. [12] proposed a Bayesian hierarchical model that jointly estimates the expected number of deaths, and the reporting delay distribution to nowcast COVID-19 fatalities in Sweden. This model offers enhanced predictive performance and flexibility by incorporating leading indicators such as the number of reported cases and COVID-19 associated ICU admissions.

A nowcasting by Bayesian smoothing approach capable of producing nowcasts in multiple disease settings was developed by [13]. This approach learns the reporting delay distribution and the time evolution of the epidemic to produce nowcasts in both stable and time-varying case reporting settings. The approach was tested on dengue in Puerto Rico and influenza-like illness (ILI) in the United States. [14] presented a framework for addressing reporting delays in malaria surveillance in Guyana. The method combines a data imputation model and network models to refine case estimates using historical data, neighboring region data, and precipitation levels. [15] introduced a Bayesian framework with sliding windows for dengue surveillance in Bangkok, Thailand, addressing reporting delays by accounting for spatial and temporal variations. A Bayesian hierarchical model for dengue nowcasting in Brazil was developed by [9]. This approach uses a Negative Binomial distribution for the reported cases with mean explained by spatial, temporal, and delay information, offering a robust correction to the reported cases. This model is used by the InfoDengue system to nowcast dengue in Brazilian municipalities [16].

The implementation of these methods are complex and requires extensive data on the historical weekly reported cases for the disease under consideration. In recent years, methods utilizing digital data, such as Google Trends indices and Twitter (now X) information on disease-related keywords, have shown notable improvement in understanding and predicting disease activity levels. These methods leverage real-time search query data to enhance the accuracy of traditional models, allowing for more timely and reliable public health responses.

For example, [17] utilized used search query logs and modeling techniques such as Elastic Net regularized regression and Gaussian Process regression to nowcast influenza-like illness in the USA. [18] used an ARIMA model augmented with Google Flu Trends data for nowcasting influenza outbreaks in the USA. They showed the incorporation of real-time search query data improves prediction accuracy compared to a model that uses only case data.

A Hidden Markov Model combining cases and Google Trends information for disease prediction was proposed by [19]. The model incorporated an autoregressive component describing case counts and a linear covariate representing Google Trends. The model was applied to predict dengue in Brazil, Mexico, Thailand, Singapore, and Taiwan, as well as influenza-like illness in the USA [20].

In [21], authors utilized Baidu search query data, which is similar to Google Trends, to nowcast hand, foot, and mouth disease across China. They utilized a meta-learning framework to dynamically select among predictive models including Principal Component Analysis, LASSO, Ridge Regression, and ARIMA. They showed the inclusion of Baidu Index data enhances prediction accuracy by providing real-time public interest metrics correlated with hand, foot, and mouth cases.

[22] used Twitter data to monitor dengue in Brazil. First, they analyzed tweet sentiments to filter tweets indicative of actual cases, and found a high correlation of the number of dengue-related tweets with official dengue data. Then, they constructed a regression model for predicting the number of dengue cases using the proportion of dengue-related tweets, and used it to develop a monitoring system that generated weekly heatmaps of dengue across cities in Brazil.

Although dengue surveillance systems in Brazil have not widely incorporated Google Trends data into predictive models, there is limited but growing evidence of its potential utility. For instance, [23] and [24] explore correlations between Google Trends and dengue and yellow fever outbreaks in São Paulo, while [25] presents a forecasting method that leverages sparse representations of Google Trends, electronic health records, and time series data across several countries, including Brazil. These developments highlight the importance of exploring alternative approaches for real-time dengue monitoring. To this end, in this paper we assess the value of Google Trends to nowcast weekly dengue cases in Brazilian states by fitting models that integrate reported dengue cases provided by the primary national tracking system InfoDengue, and Google Trends indices of dengue-related keywords. Then, we compare nowcasts produced by models that utilize only reported dengue cases, only Google Trends data, and a combination of both, with the model provided by InfoDengue [16]. As a baseline, we also use a naive approach where nowcasts are considered as the reported cases the previous week. Our aim is to evaluate the value of real-time Google Trends data in producing accurate nowcasts using simple models that do not rely on incomplete recent case data, and to determine whether these nowcasts can compete with more complex and time-consuming models.

Additionally, recognizing the importance of timely and accurate data for dengue surveillance, we developed the Dengue Tracker website (https://diseasesurveillance.github.io/dengue-tracker/). This site is updated with weekly nowcasts for each Brazilian state, and presents information through interactive maps and time trend plots to inform decision-makers and the public about dengue activity levels in real-time.

## Materials and methods

### Study region

**Fig 1:**
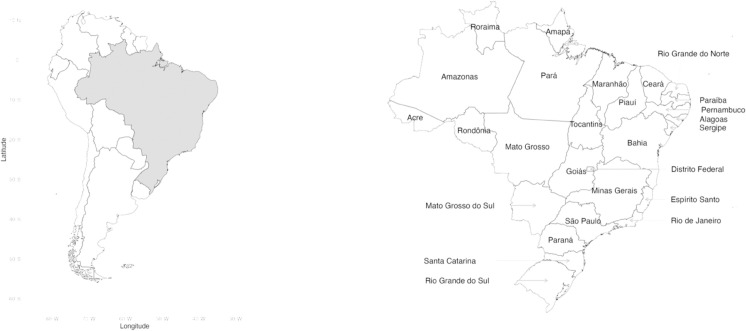
Map of South America with Brazil highlighted in gray (left), and map of the 26 states and the Federal District of Brazil (right). Administrative boundaries from South American countries obtained from Natural Earth [26] using the R package rnaturalearth [27]. Administrative boundaries of Brazilian states obtained from the Brazilian Institute of Geography and Statistics (IBGE) [28] using the R package geobr [29].

Brazil is divided into 26 states and one federal district, each with unique climatic and socio-economic characteristics that impact dengue transmission and control (Fig 1).

Several factors significantly contribute to the proliferation of dengue outbreaks in Brazil [30]. Climate change, characterized by increased temperatures, has diminished geographical barriers to dengue transmission, particularly in southern Brazil, by reducing the seasonal cold periods that typically inhibit mosquito propagation. In the Amazon region, climatic changes have made previously protected areas more susceptible to dengue outbreaks [6,7]. Rapid urbanization has led to high population densities in large cities, fostering environments conducive to mosquito breeding due to inadequate infrastructure, such as insufficient piped water and waste management systems [31]. Furthermore, high connectivity within Brazil’s urban network exacerbates the risk of disease spread [30].

**Fig 2:**
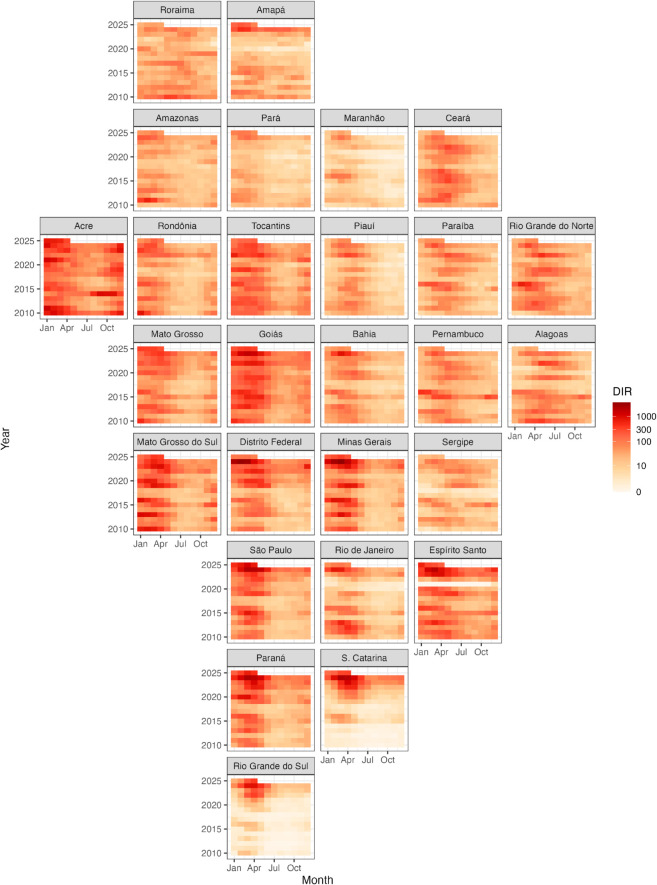
Dengue incidence rate (cases per 100k people) on a *log*_10_ scale in Brazilian states from January 2010 to April 2025, showing a seasonal pattern, with outbreaks typically occurring between January and May.

Fig 2 illustrates the seasonal pattern of monthly dengue incidence rates in Brazilian states from January 2010 to April 2025, aggregated at the state level, with data sourced from InfoDengue [32]. The figure reveals a seasonal pattern, with dengue outbreaks typically occurring from January to May. In certain regions, such as Acre, Rondônia, Mato Grosso, and Goiás in southwestern Brazil, outbreaks may even commence in November or December. The spread of the disease is generally minimal during the winter months. Moreover, it is observed that dengue outbreaks tend to be more severe every three to four years. This periodicity could be influenced by El Niño, which significantly impacts weather patterns in certain areas of Brazil [33]. Notably, the states of Acre, Espírito Santo, Goiás, Mato Grosso do Sul, Paraná, Rio Grande do Norte, São Paulo, and Tocantins experience a more substantial impact from dengue, as indicated by consistently higher incidence rates.

### Dengue case data

The InfoDengue system [34] provides comprehensive data on dengue and other arboviruses across Brazil, offering detailed insights into regional variations, trends, and severity of outbreaks. Accessible at https://info.dengue.mat.br/, the platform aggregates data provided from the Notifiable Diseases Information System (SINAN) to present dengue cases by epidemiological week and municipality. In Brazil, whenever a physician identifies a suspected dengue case, this case must be reported within a week in SINAN. Even though there are probable and laboratory confirmed cases, the InfoDengue system uses all dengue suspected cases reported in SINAN to provide early warnings as soon as possible. This is because probable and laboratory confirmed cases are prone to more sources of delay, and the likelihood of false-negative dengue results is high.

Despite being a reliable source of information, the cases shown in InfoDengue suffer from reporting delays. As mentioned in [9], although in principle dengue is meant to be reported within seven days, in practice no more than 90% of the cases are reported within 9 weeks. The difference between the provisional and final dengue cases represents the delay that needs to be accounted for in the nowcasting models. In addition, InfoDengue provides nowcasts and comprehensive visualizations into the geographic and temporal distribution of dengue, making it an essential tool for timely decision-making and proactive response to emerging outbreaks.

### Google Trends data

Google Trends (https://trends.google.com/trends/) is a tool that provides anonymized and aggregated insights into global search behaviors. The Google Trends index for a specific keyword at a given time ranges from 0 to 100, calculated by dividing the number of searches for that keyword by the total number of searches in a specific region and timeframe, enabling fair comparisons between search terms, locations, and periods.

Here, we utilize Google Trends data to understand dengue search behavior patterns that could complement official dengue data. To select the keywords for the Google Trend indices to include in the models, we calculated the Pearson correlation between country-level aggregated dengue cases and the Google Trends index for various dengue-related keywords identified in [35]. We used data from January 1, 2013, to December 31, 2023, with correlations computed using monthly resolution data, as Google Trends data cannot be obtained at a finer temporal resolution for periods exceeding five years and two months.

Google Trends indices for the keywords “sintomas dengue” (*r* = 0.93) and “dengue” (*r* = 0.90) showed the strongest correlations with dengue cases. However, the two terms were highly intercorrelated (*r* = 0.97), raising concerns about multicollinearity if both were included in the models. Additionally, the “sintomas dengue” series was sparse. Therefore, to balance predictive strength and model stability, we decided to incorporate only the Google Trends index for the keyword “dengue” into our models.

## Nowcasting methods

We evaluate the performance of several approaches for nowcasting the weekly number of dengue cases in each Brazilian state. Specifically, we use five approaches: 1) a model that uses only official dengue case data; 2) a model that uses only Google Trends data; 3) a model that integrates both official dengue case data and Google Trends indices; 4) a Bayesian nowcasting approach implemented in the InfoDengue system; and 5) a naive approach that predicts cases based on the previous week’s data.

Models are evaluated using a moving window strategy, where each model is trained on a fixed-size window of historical data. Nowcasts are then generated for the last week of the window based on this training. The window advances by one week iteratively, producing a sequence of nowcasts over time that will be compared with the actual number of cases using several error and uncertainty measures.

In this study, we began recording the weekly number of dengue cases from InfoDengue on epidemiological week 10 of 2024 (March 3). Due to reporting delays, the initially reported number of cases is considerably lower than the actual number. Each week, InfoDengue updates the case numbers, continuing this process for up to approximately 10 weeks [9]. To determine the number of weeks after which case counts can be considered complete, we computed the cumulative reporting proportions across time and states (Fig A in S1 Text). We observed that a 15-week cutoff captures the 95% reporting threshold in nearly all states – only Amapá, Minas Gerais, and Rio Grande do Sul fall slightly short, and even for those, the difference from their average delay is minimal. Based on this, we selected 15 weeks as the point beyond which reported case counts are taken to reflect the true number of cases.

We started by producing a nowcast for week 10 of 2024 (March 3, 2024), using models trained on data from the past three years, from epidemiological week 6 in 2021 to epidemiological week 6 in 2024 (February 7, 2021, to February 4, 2024), 156 weeks in total. The models were trained using data that excluded the four most recent weeks, and provided a nowcast for the current number of dengue cases (four weeks ahead). We decided to exclude the most recent four weeks of training data to balance maintaining recent information with discarding incomplete data. During this period, the reported dengue cases do not accurately reflect the actual numbers, with less than 75% of cases being reported within four weeks [9].

This procedure is repeated by moving the window forward by one week for 46 weeks, obtaining nowcasts for epidemiological weeks 10 of 2024 to 3 of 2025 (March 3rd 2024 to January 12th, 2025). By computing error metrics and uncertainty intervals over multiple windows, this approach is particularly useful for validating models in dynamic and seasonal contexts, providing robust insights into nowcasts accuracy.

Let *c*_*t*_ represent the actual number of dengue cases at week *t*, and let *y*_*t*_ represent the official number of dengue cases reported at week *t*. As previously discussed, *y*_*t*_ is lower than *c*_*t*_ due to reporting delays, and we are interested in obtaining a nowcast ${\hat{c}}_{t}$. Here, we assume the actual number of cases *c*_*t*_ is the number of dengue cases reported in InfoDengue 10 weeks after *t*. In addition, let *x*_*n*,*t*_ be the Google Trends index for the *n*th keyword at week *t*.

The nowcasting approaches considered include DC (Dengue Cases), GT (Google Trends), and DCGT (Dengue Cases and Google Trends), which use information only from dengue cases, only from Google Trends, and a combination of both datasets, respectively. These models use historical reported dengue cases *y*_*t*_ excluding the most recent weeks to produce nowcasts ${\hat{c}}_{t}$. That is, the models are trained using only data for which we expect $y_{t}\approx c_{t}$. In addition, a Bayesian nowcasting model and a naive approach are also considered. The descriptions of the five nowcasting approaches are as follows.

### DC

The DC model employs a Seasonal Autoregressive Integrated Moving-Average (SARIMA) model using dengue case data [36]. The SARIMA model is represented by a set of parameters equal to (p,d,q)×(P,D,Q,S), where *p* represents the order of auto-regression, *q* is the the order of moving-average, and *d* symbolizes differencing by which non-stationary time series are transformed into stationary time series. The time series *y*_*t*_ can be modeled as a SARIMA (p,d,q)×(P,D,Q,S) as follows:

$$\Delta^{d}\Delta_{S}^{D}y_{t}=\mu +\sum_{n=1}^{p}\phi_{n}\Delta^{d}\Delta_{S}^{D}y_{t-n}+\sum_{n=1}^{P}\Phi_{n}\Delta^{d}\Delta_{S}^{D}y_{t-Sn}+\sum_{n=1}^{q}\theta_{n}\epsilon_{t-n}\;\;+\sum_{n=1}^{Q}\Theta_{n}\epsilon_{t-Sn}+\epsilon_{t},\;\epsilon_{t}\overset{iid}{∼}𝒩\left(0,\sigma^{2}\right)$$ (1)

### GT

The GT model uses a linear model using an intercept and the Google Trends index for the keyword “dengue” as a covariate.

$$y_{t}=\mu +\sum_{i=1}^{K}\beta_{i}x_{i,t}+\epsilon_{t},\;\epsilon_{t}\overset{iid}{∼}𝒩\left(0,\sigma^{2}\right).$$ (2)

### DCGT

The DCGT model uses SARIMA with eXogenous factors (SARIMAX) [36] to combine dengue cases with Google Trends indices. The exogenous part is traditionally treated as an input unaffected by the outcome. In this application, however, Google Trends indices are driven by dengue incidence. Here, the time series *y*_*t*_ can be written as the mathematical formulation of a SARIMA (p,d,q)×(P,D,Q,S) model with Google Trends as variable *x* as follows:

$$\Delta^{d}\Delta_{S}^{D}y_{t}=\mu +\sum_{n=1}^{p}\phi_{n}\Delta^{d}\Delta_{S}^{D}y_{t-n}+\sum_{n=1}^{P}\Phi_{n}\Delta^{d}\Delta_{S}^{D}y_{t-Sn}+\sum_{n=1}^{q}\theta_{n}\epsilon_{t-n}\;\;+\sum_{n=1}^{Q}\Theta_{n}\epsilon_{t-Sn}+\sum_{i=1}^{K}\beta_{i}x_{i,t}+\epsilon_{t},\;\epsilon_{t}\overset{iid}{∼}𝒩\left(0,\sigma^{2}\right),$$ (3)

where $\Delta^{d}\Delta_{S}^{D}y_{t}=(1-B{)}^{d}(1-B{)}^{D}y_{t}$ and *B* is the back-shift operator.

### InfoDengue

InfoDengue provides nowcasts using a Bayesian hierarchical model where the observed number of events *y*_*t*,*d*,*s*_ at time *t* reported after *d* time units in spatial location *s* is assumed to follow a Negative Binomial distribution with mean $\lambda_{t,d,s}$ and dispersion *ϕ* [9]. Specifically,

$$y_{t,d,s}∼\text{NegBin}(\lambda_{t,d,s},\phi ),$$ (4)

with mean $\text{E}[y_{t,d,s}]=\lambda_{t,d,s}$ and variance $\text{Var}[y_{t,d,s}]=\lambda_{t,d,s}(1+\lambda_{t,d,s}/\phi )$. To capture the temporal and spatial variability of *y*_*t*,*d*,*s*_, the mean is expressed as

$$\log(\lambda_{t,d,s})=\mu +\alpha_{t}+\beta_{d}+\gamma_{t,d}+\eta_{w(t)}+\psi_{s}+\beta_{d,s}+X_{t,d,s}^{\prime }\delta .$$ (5)

Here, *μ* represents the overall mean on the log scale, and $X_{t,d,s}^{\prime }$ is a matrix of temporal, delay-related, and spatially varying covariates with associated parameter vector *δ*. $\alpha_{t}$ and $\beta_{d}$ capture time and delay structure means, respectively, modeled as first-order random walks. The model also includes random effects $\gamma_{t,d}$ to capture the interaction between time and delay, and a seasonal component $\eta_{w(t)}$. Finally, $\psi_{s}$ represents spatial variability, and $\beta_{d,s}$ captures how the delay structure varies across different spatial locations.

### Naive

The naive approach uses the number of cases reported in the previous week as the nowcast of week *t*:

$${\hat{c}}_{t}=y_{t-1}.$$ (6)

### Accuracy and uncertainty metrics

We assessed the performance of each nowcasting approach using several error measures. In addition, we computed the 95% and 50% coverage probabilities, which represent the proportion of times actual cases were covered by the 95% and 50% uncertainty intervals, respectively. Let ${\hat{c}}_{t}$ and *c*_*t*_ represent the predicted and actual number of dengue cases, respectively, at time *t*, where t=1,…,n. We computed evaluation metrics for the nowcasting weeks. These include the Root Mean Squared Error (RMSE), which measures the square root of the average squared differences between the predicted and actual values as

$$RMSE={\left[1/n\sum_{t=1}^{n}{\left({\hat{c}}_{t}-c_{t}\right)}^{2}\right]}^{1/2},$$ (7)

and the Root Mean Squared Percentage Error (RMSPE) as the square root of the average squared percentage errors:

$$\mathrm{RMSPE}={\left\{1/n\sum_{t=1}^{n}{\left[\left({\hat{c}}_{t}-c_{t}\right)/c_{t}\right]}^{2}\right\}}^{1/2}.$$ (8)

RMSPE is useful when the relative error is more meaningful than the absolute error, highlighting proportional discrepancies. We also computed the logarithmic score (logscore), a proper scoring rule that evaluates the full predictive distribution. Assuming $c_{t}∼𝒩({\hat{c}}_{t},\;\sigma_{t}^{2})$, where ${\hat{c}}_{t}$ is the point-nowcast and $\sigma_{t}$ its predictive standard deviation, the logscore is defined as

$$logscore\;=\;-\;\frac{1}{n}\sum_{t=1}^{n}\log\;[\frac{1}{\sigma_{t}\sqrt{2\pi }}\exp\;(-\frac{(c_{t}-{\hat{c}}_{t}{)}^{2}}{2\sigma_{t}^{2}})].$$ (9)

A lower logscore indicates a better probabilistic forecast.

### Implementation

All analyses were performed using the statistical software R version 4.4.3 [37]. Nowcasts and 95% and 50% confidence intervals for the DCGT and DC models were obtained using the function auto.arima() from the forecast package using the default maximal orders [38]. Results for the GT model were obtained with the linear model function lm() from R. For reproducibility purposes, data and code to apply these methods are provided in the GitHub repository https://github.com/diseasesurveillance/dengue-tracker/tree/main/paper.

## Results

This section presents the error and uncertainty measures obtained for each method across all states. Results are not shown for Espírito Santo as this state stopped reporting dengue cases to the federal governement since epidemiology week 16 of 2024 (April 14), resulting in missing case counts from that date onwards. Furthermore, the data for epidemiology week 24 of 2024 (June 9) was not uploaded by InfoDengue, so this week’s comparison was skipped in our analysis. For comparisons requiring this week’s data as the “true value”, the data from epidemiology week 25 of 2024 (June 16) were used.

Tables 1 to 4 present the error measures obtained for each state and nowcasting approach, where red indicates the best performance, and blue represents the second-best performance model.

**Table 1:** RMSE obtained for each state and nowcasting approach. Red and blue represent the best and the second best performances respectively (the lowest and the second lowest error).

	DCGT	DC	GT	InfoDengue	Naive
Acre	312.88	318.62	282.33	376.52	358.8
Alagoas	227.45	267.59	448.52	207.46	376.73
Amapá	147.12	140.42	114.06	190.68	287.16
Amazonas	105.42	286.71	97.82	192.3	232.48
Bahia	6039.85	10545.61	4198.09	8770.68	5148.4
Ceará	535.42	541.32	1415.7	364.29	640.09
Distrito Federal	2852.67	2520.59	1673.02	1949.51	2119.02
Espírito Santo	-	-	-	-	-
Goiás	342330	218082.08	3622.88	2651.6	5833.3
Maranhão	227.65	419.62	222.01	567.9	350.53
Mato Grosso	584.93	911.6	436.12	509.35	1093.76
Mato Grosso do Sul	493.71	546.41	500.67	1996.55	833.92
Minas Gerais	12789.48	13415.67	12942.06	10837.09	34615.34
Pará	522.67	565.1	293.79	649.33	754.16
Paraíba	384.31	616.74	158.61	292.55	339.61
Paraná	10646.04	10275.83	5109.95	3102.5	11463.52
Pernambuco	745.59	814.96	519.87	902.92	1046.99
Piauí	227.62	182.96	241.9	114.95	296.35
Rio de Janeiro	2068.78	6044.89	1903.36	8468.87	4372.11
Rio Grande do Norte	282.33	424	319.4	174.85	239.63
Rio Grande do Sul	4495.9	3520.09	3853.64	1763.9	4080.09
Rondônia	138.04	338.82	121.9	284.95	213.8
Roraima	47.38	47.74	38.72	33.73	49.23
Santa Catarina	4797.95	4931.92	6097.02	5460.02	9197.25
São Paulo	45085.82	27931.58	31150.25	37001.61	63894.79
Sergipe	73.23	122.74	123.14	430.91	135.22
Tocantins	206.23	268.35	206.64	140.32	195.09

**Table 2:** RMSPE obtained for each state and nowcasting approach. Red and blue represent the best and the second best performances respectively (the lowest and the second lowest error).

	DCGT	DC	GT	InfoDengue	Naive
Acre	0.62	0.61	0.55	0.57	2.51
Alagoas	0.52	0.72	0.36	0.66	3.53
Amapá	2.5	2.45	1.04	3.52	100.64
Amazonas	0.25	0.36	0.34	0.5	1.36
Bahia	0.46	0.6	0.43	0.38	1.53
Ceará	0.4	0.41	0.49	0.54	3.98
Distrito Federal	0.57	0.52	0.34	0.3	0.41
Espírito Santo	-	-	-	-	-
Goiás	0.49	0.55	0.29	0.36	1.19
Maranhão	0.52	0.86	0.33	1.59	10.39
Mato Grosso	0.35	0.7	0.2	0.41	1.08
Mato Grosso do Sul	0.38	0.36	0.25	0.72	2.96
Minas Gerais	2.03	2.46	0.29	3.59	7.8
Pará	1.17	2.06	0.44	0.87	5.92
Paraíba	0.43	0.83	0.15	0.24	0.54
Paraná	0.28	0.32	0.21	0.93	0.62
Pernambuco	0.36	0.65	0.24	0.44	1.7
Piauí	0.7	0.67	0.35	0.6	1.68
Rio de Janeiro	0.38	0.42	0.28	0.34	1.24
Rio Grande do Norte	0.32	0.77	0.21	0.38	0.58
Rio Grande do Sul	0.5	0.41	0.46	0.89	0.76
Rondônia	0.49	0.61	0.46	1.87	9.24
Roraima	0.45	0.45	0.52	0.43	1.15
Santa Catarina	0.34	0.34	0.44	0.68	0.74
São Paulo	0.54	0.43	0.27	0.44	1.04
Sergipe	0.25	0.47	0.33	0.54	1.42
Tocantins	0.41	0.44	0.45	0.33	0.5

**Table 3:** 95% coverage probabilities (left) and 50% coverage probabilities (right) obtained for each state and nowcasting approach. Red represent models closest to nominal coverage, with preference given to those with higher values in case of ties.

	95% coverage probabilities			50% coverage probabilities		
	DCGT	DC	GT	DCGT	DC	GT
Acre	0.93	0.93	0.84	0.53	0.51	0.53
Alagoas	0.93	0.84	1	0.42	0.58	0.67
Amapá	0.84	0.84	0.87	0.31	0.31	0.38
Amazonas	1	1	1	0.62	0.69	0.51
Bahia	0.91	0.89	0.8	0.6	0.53	0.47
Ceará	0.91	0.93	0.96	0.69	0.73	0.42
Distrito Federal	0.89	0.91	0.89	0.44	0.51	0.31
Espírito Santo	-	-	-	-	-	-
Goiás	1	0.97	0.78	0.67	0.61	0.49
Maranhão	0.93	0.84	0.91	0.62	0.56	0.64
Mato Grosso	0.91	0.82	1	0.64	0.56	0.64
Mato Grosso do Sul	1	1	1	0.78	0.8	0.53
Minas Gerais	0.82	0.84	0.98	0.38	0.47	0.51
Pará	0.84	0.93	0.89	0.51	0.67	0.29
Paraíba	0.93	0.93	0.98	0.8	0.78	0.76
Paraná	0.98	0.96	0.98	0.82	0.76	0.64
Pernambuco	0.84	0.93	1	0.67	0.62	0.67
Piauí	0.96	0.98	0.98	0.6	0.76	0.62
Rio de Janeiro	0.98	0.96	0.98	0.67	0.62	0.53
Rio Grande do Norte	0.96	0.96	1	0.64	0.67	0.64
Rio Grande do Sul	0.91	0.98	0.84	0.58	0.78	0.51
Rondônia	1	1	1	0.82	0.71	0.44
Roraima	0.93	0.93	0.96	0.67	0.69	0.6
Santa Catarina	1	1	0.8	0.69	0.78	0.42
São Paulo	0.7	0.89	0.93	0.43	0.57	0.55
Sergipe	0.95	0.93	1	0.73	0.73	0.57
Tocantins	1	1	0.93	0.55	0.61	0.48

**Table 4:** Logscore obtained for each state and nowcasting approach. Red and blue represent the best and the second best performances respectively (the lowest and the second lowest logscore).

	DCGT	DC	GT
Acre	6.82	6.81	6.83
Alagoas	6.75	7.19	6.53
Amapá	8.97	8.94	7.14
Amazonas	6.09	6.61	6.21
Bahia	9.06	9.24	11.17
Ceará	7.15	7.2	8.37
Distrito Federal	7.79	7.78	7.74
Espírito Santo	-	-	-
Goiás	8.43	8.62	8.36
Maranhão	5.81	7.06	5.79
Mato Grosso	7.61	8.54	7.04
Mato Grosso do Sul	7.34	7.5	6.89
Minas Gerais	15.23	16.99	9.46
Pará	9.6	10.6	7.59
Paraíba	7.39	8.37	6.19
Paraná	9.4	9.59	8.84
Pernambuco	8	8.31	7.15
Piauí	6.23	6.2	5.69
Rio de Janeiro	8.15	8.35	8.11
Rio Grande do Norte	6.54	7.44	6.06
Rio Grande do Sul	8.37	8.33	8.54
Rondônia	5.95	6.34	5.87
Roraima	5.15	5.16	5.09
Santa Catarina	9.07	9.16	9.84
São Paulo	12.79	11.18	10.51
Sergipe	5.61	6.29	5.84
Tocantins	6.5	6.62	6.62

Table 1 presents the RMSE values for prediction errors of each model. Overall, the GT model performed the best among the five approaches, achieving the most accurate predictions in 12 out of 26 states evaluated. The InfoDengue and DCGT models showed comparable performance, excelling in 10 and 3 states, respectively. Conversely, the DC and naive approaches performed the worst. The former one only performs the best in São Paulo, and the later one yields no advantage in any states. In several states, such as Pará, Paraíba, Pernambuco and Rio de Janeiro, the GT model considerably outperformed the second-best models (the error of the second-best performing model was more than about 1.5 times the error of the best-performing model). The smallest errors were observed in Roraima, all below 100, whereas São Paulo exhibited the largest errors, ranging from approximately 28,000 to 45,000, reflecting the unbalanced disease burden across states.

RMSPE (Table 2) values indicate that the top model remains GT, and the other models are less competitive. Across states, the ranking by decreasing number of states with lowest RMSPE is GT, followed by DCGT, then InfoDengue, DC, and finally the naive approach. For RMSPE, GT leads in 17 states, while DCGT and InfoDengue leads in 4 different states. The DC model only leads in 2 states, and the naive approach does not outperform in any states. Moreover, in many states, there is a notable difference between the performance of the best and second-best models. For example, in Alagoas, Amapá, Minas Gerais and Pará the error of the second-best performing model was more than about 1.5 times the error of the best-performing model.

As shown in Table 4, the GT model achieves the best (lowest) logscore in 18 of 26 evaluated states, making it the top performer in the majority of states. The DCGT approach comes out best in 6 states, while the DC model leads in only 2 states. Looking at second-best finishes, DCGT is runner-up in 16 cases, DC in 8, and GT in 3. These results indicate that a pure Google Trends signal tends to yield the most accurate nowcasts overall, with the other two time series models performing only modestly across states.

**Fig 3:**
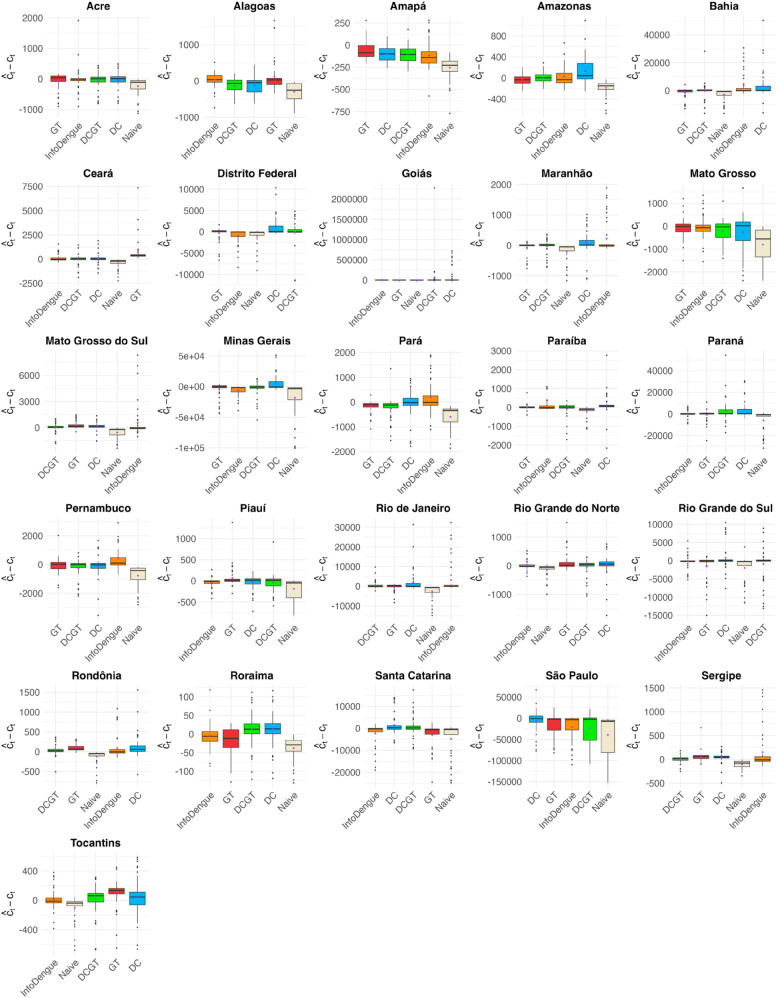
Boxplots of differences between the nowcasts and actual dengue cases in Brazilian states. Models are sorted in ascending order of their mean absolute difference. Box colors represent different models. Black dots represent outliers in the distribution of differences between the nowcasts and the actual case counts, and purple dots indicate the mean difference.

**Fig 4:**
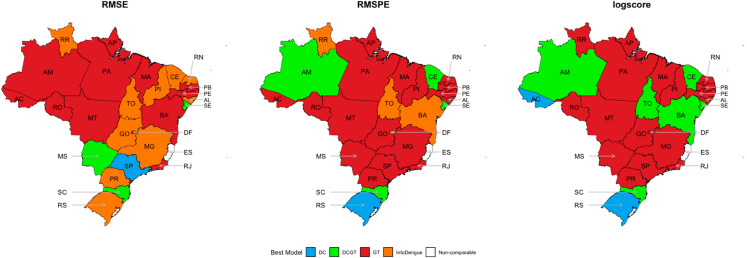
Map of Brazilian states indicating the top-performing nowcasting models based on selected evaluation metrics, with GT and InfoDengue performing best overall. Administrative boundaries of Brazilian states obtained from the Brazilian Institute of Geography and Statistics (IBGE) [28] using the R package geobr [29].

Fig 3 displays boxplots of the differences between the nowcasts and the actual number of dengue cases across Brazilian states. Models are sorted by their mean absolute error, from lowest to highest, allowing for a clear comparison of performance. Fig 4 complements this by providing a visual summary of the top-performing nowcasting models across Brazilian states, based on multiple evaluation metrics, highlighting GT and InfoDengue as the best methods overall.

Table 3 displays the 95% and 50% coverage probabilities obtained for each state and nowcasting approach. In these tables, results for InfoDengue and the naive approach are not included. For InfoDengue, nowcasts for this method are provided at municipality level, while our analysis is conducted at the state level. We cannot simply aggregate uncertainty intervals from InfoDengue to make the comparison. As to the naive approach, this simply uses as nowcasts the reported cases from last week, and does not provide any intervals. For the 95% coverage probabilities, we found that all three models exhibited high coverage rates which are around 95% for some states, if the best model according to this criterion is considered. The best performance model is GT, achieving the highest coverage in 17 different states. DCGT model and DC model achieved the highest coverage in 11 and 10 states respectively.

For the 50% coverage rate, model DC and DCGT yield better coverage probabilities. They have the best coverage rate in 13 and 10 states respectively. GT only obtains the best performance in seven states. However, across most states, the empirical 50% coverage rates slightly exceed the nominal 50%, suggesting that the 50% prediction intervals are somewhat conservative.

**Fig 5:**
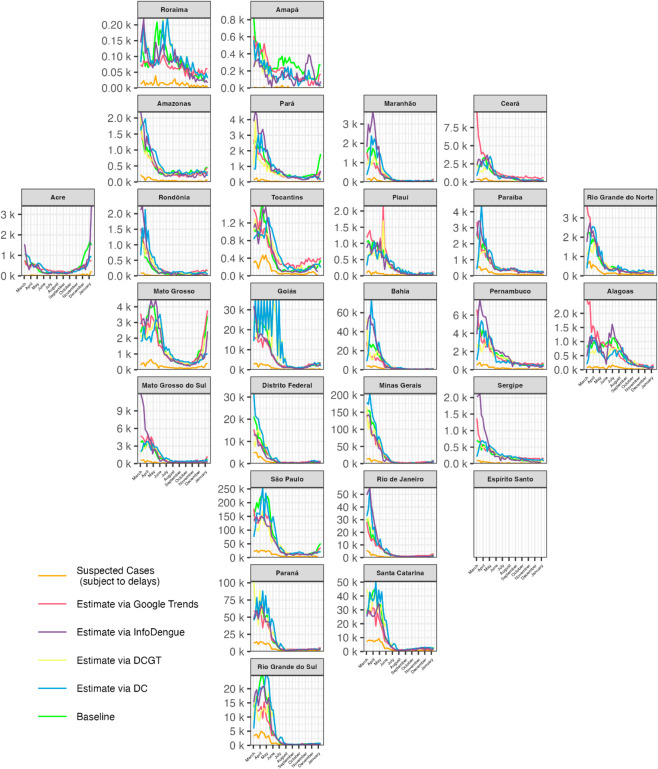
Model predictions for epidemiological weeks 10 of 2024 to 3 of 2025 (March 2024 to January 2025). The green line represents the true number of cases (reported after a 15-week delay) used as the benchmark, while the orange line shows the suspected cases that are reported each week, reflecting reporting delays. Overall, GT and InfoDengue provide the most accurate forecasts, staying closest to the green line compared to DC and DCGT. Goiás predictions were capped to 35,000 cases to mitigate the impact of overfitting observed on subplot in the DC and DCGT models for several weeks.

Fig 5 shows the weekly nowcasting results for each method. The green lines represent the reported cases for a given epidemiological week after 15 weeks. We consider these values as the true number of cases to benchmark the models’ performance. Notably, the orange lines representing the number of suspected cases reported each week are consistently the lowest, serving as a practical “lower bound”, with various models employed to adjust this lower limit. Overall, the GT nowcasts (red lines) align closely with the true values in many regions – for example, in Amazonas, Acre, and Rio de Janeiro – while the InfoDengue forecasts (purple lines) tend to perform particularly well in several others, such as Ceará, Paraná, and Rio Grande do Sul. Both approaches capture the general temporal trends with good accuracy and each outperforms the other in roughly half of the states. By contrast, the DCGT model still adds value in certain areas (e.g. Amazonas, Acre, Mato Grosso do Sul), and the DC forecasts can appear more variable, making them somewhat less reliable overall.

## Dengue Tracker website

Timely and accurate information on dengue cases is crucial for prevention and control. We developed the Dengue Tracker website (https://diseasesurveillance.github.io/dengue-tracker/) to provide weekly updates on the number of official dengue cases per state in Brazil, as well as at the country level. Additionally, the website provides corrected case counts incorporating information from Google Trends (using our GT model) and also the InfoDengue results. We believe these reports will assist policymakers in understanding dengue levels and guide their decisions.

Each week, the number of dengue cases is downloaded through InfoDengue’s API, and the Google Trends information for the specific keywords is downloaded from https://trends.google.com/trends/. Data are download from the last 5 years up to the week we are interested in nowcasting. At the country level, Dengue Tracker shows the dengue incidence rate through an interactive choropleth map. Besides, it depicts the time series of the number of cases, the fitted model, and the corrections from both our model and InfoDengue’s for each state in a plot with the shape of Brazil. Reports are also provided for each state. The website is built using RMarkdown [39] and GitHub Pages. The graphic components are built using ggplot2 [40], plotly [41], geofacet [42] and leaflet [43].

## Discussion

Reporting delays in surveillance data make traditional surveillance systems ineffective for planning and control. In this paper, we compared the usefulness of integrating Google Trends information for dengue nowcasting in Brazil with other analytical approaches that rely solely on reported data. Specifically, we evaluated the error and uncertainty produced by approaches that used only Google Trends information (GT), only dengue case data (DC), and a combination of both (DCGT). In addition, we compared these results with the nowcasting algorithm provided in InfoDengue and a naive approach, where the number of cases in a given week was nowcasted as the number of cases reported in the previous week.

Our study demonstrates the effectiveness of combining Google Trends information with reported case data for nowcasting dengue in Brazil. We show that using the reported number of cases as the nowcast for the following week, as done in the naive approach, is insufficient for real-time monitoring since it considerably underestimates the actual number of dengue cases. Overall, the GT model demonstrates the lowest error across most states, outperforming other models in all metrics, with InfoDengue ranking second. In contrast, neither DCGT nor DC provides notable improvements in nowcasting accuracy. The results, as illustrated through boxplots and accuracy metrics, confirm that incorporating Google Trends data effectively enhances predictive performance.

We could not provide comprehensive comparisons across all models and measures due to fundamental methodological differences. The naive model produces only point predictions without uncertainty bounds, while InfoDengue provides municipality-level nowcasts that cannot be meaningfully aggregated to state-level comparisons. Despite these constraints, our GT model demonstrated competitive point prediction performance against both models where direct comparisons were feasible.

In our analyses, we initially selected a moving window of 3 years (from 2021-02-07 to 2024-02-04) to ensure sufficient training data while excluding anomalous dynamics during the early COVID-19 period in 2020. We also conducted a sensitivity analysis using alternative window sizes of 1 year (from 2023-02-05 to 2024-02-04) and 2 years (from 2022-02-06 to 2024-02-04). These results are presented in S2 Text. We observed that the performance of DC and DCGT models is more sensitive to changes in the training window size. However, this variation does not alter the overall conclusions of the study, as the relative model performance and key findings remain consistent across all tested window sizes.

We also assessed the performance metrics for several nowcast horizons: the current week, one week prior, two weeks prior, and three weeks prior. The results indicate that InfoDengue’s errors shrink steadily as we move from the current week to one, two, and three weeks back – its nowcasts for older weeks are more accurate than for the most recent week. The other models do not show this pattern. This may happen because InfoDengue leverages the full reporting-delay distribution (including the most recent four weeks), while the other models omit those weeks and therefore cannot capture the same delay dynamics. These results are shown in S3 Text.

In our study, we excluded Espírito Santo from model comparisons because the state ceased reporting dengue cases. However, it is possible to fit an appropriate GT model using historical data and continue nowcasting with Google Trends data, even after reporting has stopped — this approach was actually implemented on the Dengue Tracker website. Another noteworthy case is Rio de Janeiro, where changes in the notification system’s infrastructure and workflow were introduced during the epidemic to achieve a faster response. This shift was not captured by InfoDengue, leading to nowcasts that considerably overestimated the actual number of cases from March to April, whereas GT’s nowcasts were much closer to the true figures. In these situations, our Google Trends-only model continued to produce timely estimates and was even adopted by the Ministry of Health in Rio de Janeiro when the official feeds failed. Although the Google Trends model sometimes outperforms and sometimes underperforms the other approaches, that variability highlights the need for multiple, complementary nowcasting methods rather than reliance on a single system.

One limitation of our study is that Google Trends data is inherently biased since not all individuals use Google to search for dengue-related information. This bias may result in underrepresentation of specific populations or regions, potentially affecting the accuracy of our models. Further research is needed to understand how different population groups use Google to search for dengue information, how to select dengue-related keywords that accurately reflect disease transmission, and to develop models that integrate Google Trends and dengue case data in the most effective way.

Our models rely on assumptions of normality and homoscedasticity, which are not fully satisfied in all cases. Nevertheless, the models exhibited strong predictive performance, as well as trustworthy uncertainty quantification. Developing models with more flexible error structures remains an important direction for future research. Moreover, in this study, we employed statistical models that excluded the most recent weeks of incomplete information to generate dengue nowcasts. Although this approach allowed us to demonstrate the superior performance of approaches using Google Trends data compared to models relying only on reported cases, further work could be done to develop models that utilize incomplete data to further improve predictive accuracy, although this would come at the cost of relying on more data sources than the current GT model.

In addition, the models used in this study are limited in their ability to detect sudden changes in dengue incidence, since they heavily rely on historical data. This limitation hinders their effectiveness in identifying abrupt outbreaks or sharp increases in cases. Future research will explore more flexible approaches to improve responsiveness. Additionally, we plan to investigate the incorporation of variables like climate and socio-economic factors, known to influence dengue transmission, into future models. Moreover, we intend to develop spatial models that allow us to obtain nowcasts at finer geographical resolutions, such as microgregion or municipality levels [44]. This enhancement will provide localized nowcasts, enabling more precise public health interventions.

Effective and timely communication of dengue activity levels is crucial for planning and response efforts in public health. To address this need, we also developed Dengue Tracker (https://diseasesurveillance.github.io/dengue-tracker/index.html), a system designed to aggregate, analyze, and visualize dengue case data. This system provides weekly nowcasts at the state level in Brazil, aiding decision-makers and the public in understanding current risk levels. The Dengue Tracker website is updated weekly and features interactive maps and time series plots that dynamically present the latest dengue information across Brazil. By integrating our nowcasting models that use Google Trends information into this platform, the website delivers real-time alerts and trend analyses for better disease prevention and control.

In conclusion, our study presents a promising approach to improving dengue surveillance in Brazil by leveraging Google Trends data, which relies on an entirely different source of information than official case reports. This allows the model to remain functional even when official data are delayed or unavailable. Rather than replacing traditional models, our approach offers a valuable complement, helping ensure that surveillance does not depend on a single source of information. This enhances situational awareness for public health authorities and the general public, facilitating timely responses to changes in dengue activity and ultimately helping to reduce the impact of dengue and improve population health.

## Supporting information

S1 Text Cumulative reporting proportions across time and states.
(PDF)

S2 Text Sensitivity analysis for different length of time-windows.
(PDF)

S3 Text Performance metrics for different nowcast horizons.
(PDF)
