# Supplementary material for: Dengue nowcasting in Brazil by combining official surveillance data and Google Trends information
Source: PLoS Negl Trop Dis. 2025 Aug 18;19(8):e0012501. doi: 10.1371/journal.pntd.0012501 (PMC12373277; doi:10.1371/journal.pntd.0012501)
Supplement: S1 Text — Cumulative reporting proportions across time and states. (PDF) [file pntd.0012501.s001.pdf]

1 Cumulative reporting proportions across time and states

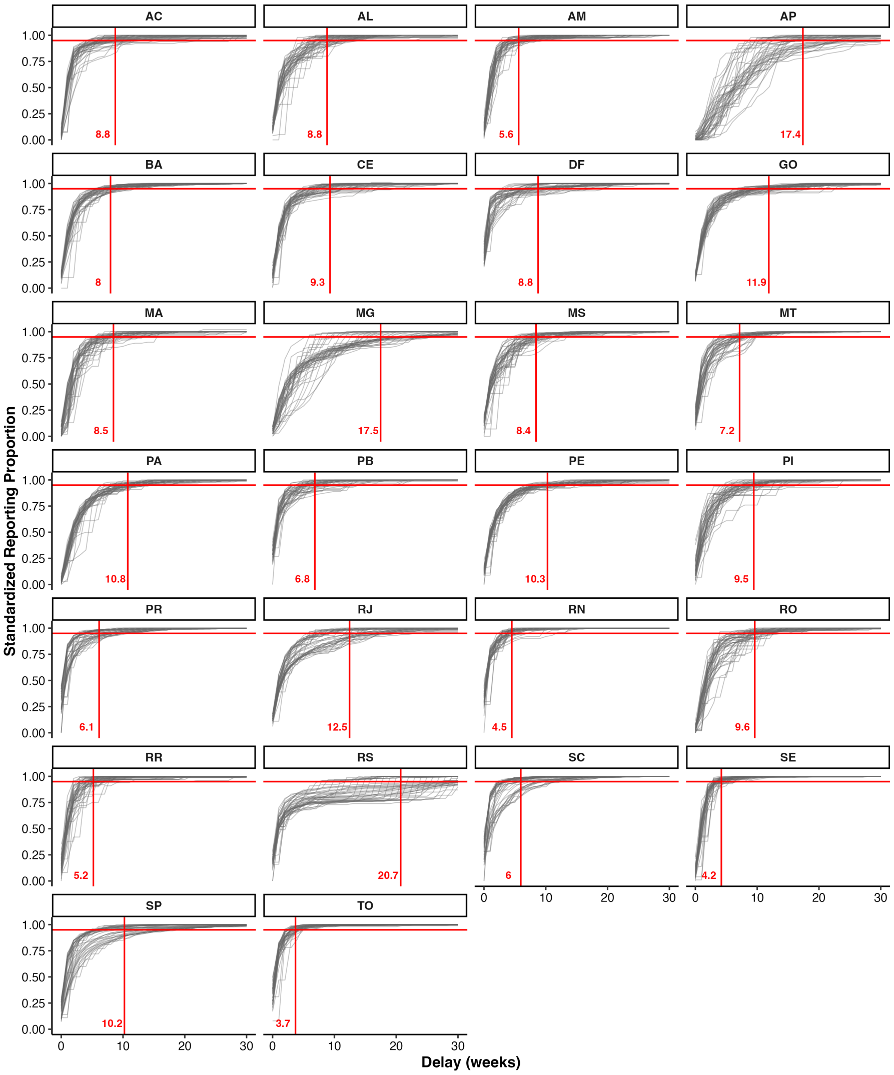

Fig A: Normalized cumulative reporting proportions for all Brazilian states except Espírito Santo, covering epidemiological weeks 10 to 52 in 2024. Each grey line depicts the cumulative proportion of cases reported by delay d weeks relative to the total reported by 30 weeks (i.e. reported cases with delay d divided by the reported cases by week 30). In each facet, a solid red vertical line marks the state-specific average 95% quantile delay (mean week at which 95% of cases are reported). The horizontal red line at 0.95 denotes the 95% reporting threshold.
