# Supplementary material for: Dengue nowcasting in Brazil by combining official surveillance data and Google Trends information
Source: PLoS Negl Trop Dis. 2025 Aug 18;19(8):e0012501. doi: 10.1371/journal.pntd.0012501 (PMC12373277; doi:10.1371/journal.pntd.0012501)
Supplement: S2 Text — Sensitivity analysis for different length of time-windows. (PDF) [file pntd.0012501.s002.pdf]

1 Sensitivity analysis for different length of time-windows

Performance metrics for the models evaluated under two training windows: a two-year window (left) covering February 7, 2022, to February 4, 2024, and a one-year window (right) spanning February 7, 2023, to February 4, 2024. In both cases, models generate weekly nowcasts over 46 epidemiological weeks, from week 10 of 2024 to week 3 of 2025 (March 3, 2024, to January 12, 2025).

	DCGT	DC	GT	InfoDengue	Naive
Acre	307.86	336.77	273.81	376.52	358.8
Alagoas	215.64	251.48	465.91	207.46	376.73
Amapá	191.21	154.12	106.3	190.68	287.16
Amazonas	122.97	435.51	88.66	192.3	232.48
Bahia	20588.3	5916.4	4201.02	8770.68	5148.4
Ceará	488.14	677.17	1427.46	364.29	640.09
Distrito Federal	3505.77	2708.13	1673.12	1949.51	2119.02
Espírito Santo	-	-	-	-	-
Goiás	3145.85	5748.16	3190.55	2651.6	5833.3
Maranhão	270.02	283.62	229.84	567.9	350.53
Mato Grosso	630.86	945.32	455.42	509.35	1093.76
Mato Grosso do Sul	521.16	576.07	513.3	1996.55	833.92
Minas Gerais	33703.84	456827.89	13088.85	10837.09	34615.34
Pará	513.86	529.04	293.81	649.33	754.16
Paraíba	395.79	483.96	162.34	292.55	339.61
Paraná	9088.72	14356.9	5171.27	3102.5	11463.52
Pernambuco	787.69	823.69	842.49	902.92	1046.99
Piauí	226.96	196	221.94	114.95	296.35
Rio de Janeiro	1714.71	16268.96	1961.04	8468.87	4372.11
Rio Grande do Norte	253.98	446.44	321.01	174.85	239.63
Rio Grande do Sul	4197.17	5377.94	3878.29	1763.9	4080.09
Rondônia	158.71	198.19	129.56	284.95	213.8
Roraima	46.17	55.83	38.2	33.73	49.23
Santa Catarina	7706.04	7730.11	6125.72	5460.02	9197.25
São Paulo	39821.11	53588.35	31607.54	37001.61	63894.79
Sergipe	73.84	117.4	129.67	430.91	135.22
Tocantins	218.33	271.17	231.91	140.32	195.09

	DCGT	DC	GT	InfoDengue	Naive
Acre	307.45	440.81	291.58	376.52	358.8
Alagoas	270.83	317.56	312.21	207.46	376.73
Amapá	169.15	160.87	93.58	190.68	287.16
Amazonas	148.15	2475.91	103.57	192.3	232.48
Bahia	22028.43	44379.38	4226.26	8770.68	5148.4
Ceará	481.07	838.52	824.77	364.29	640.09
Distrito Federal	3807.14	3166.66	1666.41	1949.51	2119.02
Espírito Santo	-	-	-	-	-
Goiás	6295.51	31810.06	3229.08	2651.6	5833.3
Maranhão	246.53	603.51	244.27	567.9	350.53
Mato Grosso	603.92	974.74	506.35	509.35	1093.76
Mato Grosso do Sul	364.78	1317.02	435.82	1996.55	833.92
Minas Gerais	13429.71	930029.67	13404.39	10837.09	34615.34
Pará	442.85	861.07	288.85	649.33	754.16
Paraíba	480.03	62896.9	278.84	292.55	339.61
Paraná	17467.71	20182.31	5366.66	3102.5	11463.52
Pernambuco	2145.89	36082.5	752.85	902.92	1046.99
Piauí	220.75	471.45	178.14	114.95	296.35
Rio de Janeiro	3878.28	40424440.41	2048.91	8468.87	4372.11
Rio Grande do Norte	315.35	2347.9	203.55	174.85	239.63
Rio Grande do Sul	3966.36	13064.75	4036.57	1763.9	4080.09
Rondônia	126.1	338.76	79.26	284.95	213.8
Roraima	43.13	50.03	37.34	33.73	49.23
Santa Catarina	9287.54	12282.83	6056.62	5460.02	9197.25
São Paulo	38204.79	432405.77	33072.75	37001.61	63894.79
Sergipe	67.28	117.05	55.55	430.91	135.22
Tocantins	194.41	430.28	227.05	140.32	195.09

Table A: RMSE obtained for each state and nowcasting approach. Red and blue represent the best and the second best performances respectively.

	DCGT	DC	GT	InfoDengue	Naive
Acre	0.62	0.67	0.54	0.57	2.51
Alagoas	0.63	0.84	0.74	0.66	3.53
Amapá	6.89	10.96	0.43	3.52	100.64
Amazonas	0.24	0.43	0.42	0.5	1.36
Bahia	0.65	2.03	0.42	0.38	1.53
Ceará	0.42	0.56	0.41	0.54	3.98
Distrito Federal	1.03	1.04	0.38	0.3	0.41
Espírito Santo	-	-	-	-	-
Goiás	0.54	0.64	0.26	0.36	1.19
Maranhão	0.74	0.72	0.54	1.59	10.39
Mato Grosso	0.35	0.64	0.26	0.41	1.08
Mato Grosso do Sul	0.32	0.5	0.2	0.72	2.96
Minas Gerais	1.15	3.13	0.28	3.59	7.8
Pará	2.21	3.35	0.4	0.87	5.92
Paraíba	0.65	1.1	0.31	0.24	0.54
Paraná	0.3	0.79	0.19	0.93	0.62
Pernambuco	0.42	0.82	0.42	0.44	1.7
Piauí	0.66	0.8	0.39	0.6	1.68
Rio de Janeiro	0.66	0.64	0.41	0.34	1.24
Rio Grande do Norte	0.52	0.9	0.3	0.38	0.58
Rio Grande do Sul	0.54	0.9	0.5	0.89	0.76
Rondônia	0.64	0.89	0.49	1.87	9.24
Roraima	0.5	0.5	0.44	0.43	1.15
Santa Catarina	0.4	0.58	0.31	0.68	0.74
São Paulo	0.41	0.59	0.31	0.44	1.04
Sergipe	0.26	0.46	0.24	0.54	1.42
Tocantins	0.36	0.56	0.49	0.33	0.5

	DCGT	DC	GT	InfoDengue	Naive
Acre	0.69	0.66	0.51	0.57	2.51
Alagoas	0.53	0.57	0.44	0.66	3.53
Amapá	3.79	3.43	0.7	3.52	100.64
Amazonas	0.26	0.36	0.21	0.5	1.36
Bahia	0.64	0.54	0.42	0.38	1.53
Ceará	0.36	0.43	0.44	0.54	3.98
Distrito Federal	0.74	0.62	0.38	0.3	0.41
Espírito Santo	-	-	-	-	-
Goiás	0.39	0.4	0.28	0.36	1.19
Maranhão	0.49	0.63	0.5	1.59	10.39
Mato Grosso	0.35	0.55	0.22	0.41	1.08
Mato Grosso do Sul	0.48	0.47	0.25	0.72	2.96
Minas Gerais	2.34	2.23	0.29	3.59	7.8
Pará	1.91	2.03	0.44	0.87	5.92
Paraíba	0.48	0.8	0.23	0.24	0.54
Paraná	0.37	0.62	0.23	0.93	0.62
Pernambuco	0.4	0.66	0.29	0.44	1.7
Piauí	0.82	0.47	0.39	0.6	1.68
Rio de Janeiro	0.41	0.47	0.34	0.34	1.24
Rio Grande do Norte	0.29	0.63	0.2	0.38	0.58
Rio Grande do Sul	0.69	0.63	0.52	0.89	0.76
Rondônia	0.62	0.82	0.44	1.87	9.24
Roraima	0.88	0.76	0.48	0.43	1.15
Santa Catarina	0.54	0.47	0.36	0.68	0.74
São Paulo	0.46	2.12	0.27	0.44	1.04
Sergipe	0.29	0.5	0.32	0.54	1.42
Tocantins	0.4	0.46	0.42	0.33	0.5

Table B: RMSPE obtained for each state and nowcasting approach. Red and blue represent the best and the second best performances respectively.

	DCGT	DC	GT
Acre	0.84	0.84	0.82
Alagoas	0.84	0.91	0.42
Amapá	0.89	0.89	1
Amazonas	0.98	0.98	0.91
Bahia	0.91	0.93	0.73
Ceará	0.91	0.91	0.96
Distrito Federal	0.89	0.91	0.91
Espírito Santo	-	-	-
Goiás	0.95	1	0.8
Maranhão	0.91	0.91	0.93
Mato Grosso	0.91	0.87	0.82
Mato Grosso do Sul	1	1	1
Minas Gerais	0.87	0.64	0.98
Pará	0.87	0.93	0.87
Paraíba	0.96	0.95	0.89
Paraná	0.96	0.91	0.98
Pernambuco	0.96	0.9	0.76
Piauí	0.91	0.91	0.84
Rio de Janeiro	0.98	0.9	0.98
Rio Grande do Norte	0.93	0.91	0.93
Rio Grande do Sul	0.91	0.93	0.82
Rondônia	0.98	0.96	0.98
Roraima	0.93	0.93	0.96
Santa Catarina	0.89	1	0.8
São Paulo	0.89	1	0.73
Sergipe	0.95	0.95	0.89
Tocantins	0.91	0.86	0.95

	DCGT	DC	GT
Acre	0.87	0.89	0.84
Alagoas	0.91	0.93	0.98
Amapá	0.69	0.76	0.91
Amazonas	0.98	0.98	1
Bahia	0.89	0.96	0.78
Ceará	0.93	0.96	0.96
Distrito Federal	0.84	0.89	0.87
Espírito Santo	-	-	-
Goiás	0.98	1	0.78
Maranhão	0.87	0.96	0.87
Mato Grosso	0.87	0.78	0.96
Mato Grosso do Sul	1	1	1
Minas Gerais	0.71	0.62	0.98
Pará	0.89	0.91	0.87
Paraíba	0.93	0.96	0.96
Paraná	0.93	0.98	0.98
Pernambuco	0.87	0.96	0.98
Piauí	0.96	1	0.98
Rio de Janeiro	0.93	0.95	0.96
Rio Grande do Norte	0.96	0.96	0.98
Rio Grande do Sul	0.89	0.98	0.84
Rondônia	0.98	0.98	0.93
Roraima	0.91	0.89	0.96
Santa Catarina	1	1	0.8
São Paulo	0.75	0.84	0.89
Sergipe	0.95	0.93	0.98
Tocantins	0.98	0.98	0.98

Table C: 95% coverage probabilities obtained for each state and nowcasting approach. Red represent models closest to nominal coverage.

	DCGT	DC	GT
Acre	0.44	0.42	0.44
Alagoas	0.51	0.42	0.31
Amapá	0.27	0.36	0.53
Amazonas	0.47	0.58	0.42
Bahia	0.58	0.64	0.4
Ceará	0.62	0.67	0.49
Distrito Federal	0.56	0.51	0.36
Espírito Santo	-	-	-
Goiás	0.67	0.52	0.38
Maranhão	0.67	0.62	0.6
Mato Grosso	0.58	0.44	0.33
Mato Grosso do Sul	0.76	0.64	0.6
Minas Gerais	0.42	0.42	0.53
Pará	0.58	0.6	0.24
Paraíba	0.71	0.69	0.42
Paraná	0.71	0.69	0.69
Pernambuco	0.69	0.62	0.47
Piauí	0.47	0.53	0.6
Rio de Janeiro	0.49	0.36	0.4
Rio Grande do Norte	0.62	0.6	0.47
Rio Grande do Sul	0.53	0.49	0.4
Rondônia	0.6	0.67	0.62
Roraima	0.64	0.69	0.67
Santa Catarina	0.64	0.69	0.29
São Paulo	0.66	0.55	0.34
Sergipe	0.5	0.8	0.43
Tocantins	0.59	0.36	0.52

	DCGT	DC	GT
Acre	0.42	0.49	0.53
Alagoas	0.51	0.67	0.56
Amapá	0.13	0.29	0.44
Amazonas	0.67	0.62	0.53
Bahia	0.62	0.62	0.38
Ceará	0.6	0.49	0.49
Distrito Federal	0.33	0.51	0.27
Espírito Santo	-	-	-
Goiás	0.38	0.42	0.27
Maranhão	0.56	0.76	0.62
Mato Grosso	0.6	0.58	0.44
Mato Grosso do Sul	0.8	0.76	0.42
Minas Gerais	0.29	0.47	0.47
Pará	0.51	0.6	0.22
Paraíba	0.82	0.82	0.53
Paraná	0.69	0.49	0.6
Pernambuco	0.71	0.78	0.64
Piauí	0.64	0.69	0.6
Rio de Janeiro	0.42	0.58	0.42
Rio Grande do Norte	0.62	0.71	0.6
Rio Grande do Sul	0.49	0.47	0.51
Rondônia	0.6	0.56	0.38
Roraima	0.62	0.69	0.56
Santa Catarina	0.47	0.58	0.36
São Paulo	0.5	0.52	0.57
Sergipe	0.48	0.59	0.45
Tocantins	0.64	0.61	0.43

Table D: 50% coverage probabilities obtained for each state and nowcasting approach. Red represent models closest to nominal coverage.

	DCGT	DC	GT
Acre	6.85	7.25	7.18
Alagoas	8.36	8.79	13.99
Amapá	12.18	24.54	5.94
Amazonas	5.99	6.84	6.42
Bahia	9.04	12.06	15.84
Ceará	7.24	7.66	7.12
Distrito Federal	9.25	8.45	7.61
Espírito Santo	-	-	-
Goiás	8.76	8.9	9.86
Maranhão	5.97	6.67	5.8
Mato Grosso	7.63	8.13	7.13
Mato Grosso do Sul	6.94	7.47	6.55
Minas Gerais	12.32	26.25	9.58
Pará	11.92	11.34	7.99
Paraíba	7.56	10.6	6.5
Paraná	9.48	10.13	8.83
Pernambuco	7.55	8.78	8.44
Piauí	6.3	6.57	6.28
Rio de Janeiro	8.32	9.21	8.81
Rio Grande do Norte	7.31	8.8	6.14
Rio Grande do Sul	8.22	8.78	8.73
Rondônia	5.87	6.3	Inf
Roraima	5.17	5.29	5.04
Santa Catarina	9.26	9.37	10.01
São Paulo	11.54	11.67	11.61
Sergipe	5.5	6.1	5.83
Tocantins	6.15	6.54	6.25

	DCGT	DC	GT
Acre	6.94	6.93	6.88
Alagoas	6.7	6.78	6.74
Amapá	14.05	14.05	6.41
Amazonas	6.15	6.71	6.14
Bahia	8.8	8.79	13.25
Ceará	6.97	7.2	7.84
Distrito Federal	8.34	8.13	7.93
Espírito Santo	-	-	-
Goiás	8.77	8.9	9.77
Maranhão	6.06	6.61	6.11
Mato Grosso	7.71	8.41	6.97
Mato Grosso do Sul	7.47	7.6	6.88
Minas Gerais	22.33	23.42	9.58
Pará	11.11	10.26	7.87
Paraíba	7.23	8.29	5.98
Paraná	9.36	9.73	9.03
Pernambuco	7.95	8.57	7.19
Piauí	6.02	5.91	5.96
Rio de Janeiro	8.24	8.51	8.42
Rio Grande do Norte	6.35	7.16	6.05
Rio Grande do Sul	8.47	8.62	8.52
Rondônia	6.22	6.33	5.82
Roraima	5.68	5.66	5.11
Santa Catarina	9.06	9.2	9.7
São Paulo	11.74	13.78	10.67
Sergipe	5.62	6.29	5.79
Tocantins	6.37	6.59	6.53

Table E: Logscore obtained for each state and nowcasting approach. Red and blue represent the best and the second best performances respectively (the lowest and the second lowest logscore).
