# Supplementary material for: Dengue nowcasting in Brazil by combining official surveillance data and Google Trends information
Source: PLoS Negl Trop Dis. 2025 Aug 18;19(8):e0012501. doi: 10.1371/journal.pntd.0012501 (PMC12373277; doi:10.1371/journal.pntd.0012501)
Supplement: S3 Text — Performance metrics for different nowcast horizons. (PDF) [file pntd.0012501.s003.pdf]

1 Performance metrics for different nowcast horizons

Performance metrics for previous one week (left), two weeks (middle), and three weeks (right). Model trained with 3-years time window length (the first model is trained with window from 2021-2-7 to 2024-2-4). Models generate weekly nowcasts over 46 epidemiological weeks, from week 10 of 2024 to week 3 of 2025 (March 3, 2024, to January 12, 2025).

	Past one week					Past two weeks					Past three weeks				
	DCGT	DC	GT	InfoDengue	Naive	DCGT	DC	GT	InfoDengue	Naive	DCGT	DC	GT	InfoDengue	Naive
Acre	255.06	260.06	255.84	181.32	217.14	184.88	187.84	213.73	109.05	168.48	121.58	119.76	188.95	85.19	127.41
Alagoas	216.18	251.32	468.71	152.05	272.57	190.81	238.83	532.89	120.21	224.79	187.48	205.89	502.99	101.76	197.71
Amapá	178.77	171.58	132.58	224.46	289.36	213.41	207.58	150.3	249.28	267.83	240.32	235.79	165.17	240.83	240.75
Amazonas	117.68	233.47	103.8	92.1	130	115.96	176.82	97.67	47.22	118.55	154.48	109.72	108.22	50.91	113.43
Bahia	4809.48	6825.72	3995.87	2864.15	3226.29	3374.25	4018.16	3864.01	1240.73	2530.31	2587.07	1889.35	3716.1	847.7	2219.83
Ceará	463.26	519.34	1439.6	152.39	413.97	400.37	422.88	1457.85	120.94	339.5	305.81	294.73	1345.95	112.5	325.22
Distrito Federal	2863.28	1331.21	2051.51	1904.35	1662.16	2896.14	1042.95	2139.1	1537.24	1809.35	2435.36	1291.81	2010.47	1326.03	1650.18
Espírito Santo	-	-	-	-	-	-	-	-	-	-	-	-	-	-	-
Goiás	5166555.31	178621.42	3555.92	2735.16	3950.88	797712278.49	139720.57	3679.77	2637.92	2919.22	355368.75	100395.68	3471.14	2084.84	2342.92
Maranhão	204.38	317.46	227.44	252.04	227.44	190.55	244.88	213.97	111.22	185.55	141.57	150.47	182.71	55.58	167.27
Mato Grosso	508.69	749.07	481.24	284.89	673.41	417.14	559.18	434.6	254.78	487.06	375.43	430.46	422.58	222.23	403.47
Mato Grosso do Sul	433.07	448.73	490.02	1152.53	491.88	365.91	379.76	578.39	685.34	349.78	280.64	292.67	615.05	470.27	294.47
Minas Gerais	15377.81	6001.04	14499.55	20258.22	27299.36	17016.58	12129.99	14961.54	20941.86	23221.35	19617.89	17763.16	15088.87	18649.36	20035.15
Pará	636.6	439.53	241.02	236.95	484.34	469.14	396.22	244.26	127.31	359.21	447.8	322.42	224.45	139.13	305.73
Paraíba	392.01	510.88	158.83	82.95	299.97	316.83	364.98	270.73	114.58	285.46	234.39	249.71	256.33	119.95	270.16
Paraná	10377.09	7104.51	5234.3	3990.37	7711.7	7451.54	4387.9	5399.06	4026.43	5810.68	6308.23	3931.74	5351.74	3322.08	4996.68
Pernambuco	696.09	763.16	608.47	343.94	725.27	636.27	623.18	565.72	203.78	613.5	536.37	503.04	537.11	159.63	542.15
Piauí	208.33	172.17	247.49	121.26	190.55	161.98	168.62	238.9	89.29	157.76	142	135.68	152.35	62.92	131.45
Rio de Janeiro	1887.67	3794.02	2057.7	2833.65	3238.47	2163.16	2555.23	2721.43	895.15	2903.95	2641.86	2136.68	2439.2	1400.24	2801.62
Rio Grande do Norte	247.91	352.26	367.88	60.49	174.27	203.64	270.03	387.24	50.6	189.96	156.82	170.07	379.18	58.73	190.06
Rio Grande do Sul	3711.62	2593.05	3799.26	1949.14	2874.37	3055.37	1926.47	3669.36	1731.53	2443.47	2245.23	1429.5	3465.46	1422.97	1950.55
Rondônia	137.45	241.45	134.87	54.68	140.17	114.99	169.63	143.81	59.96	131.06	70.54	87.89	169.23	61.11	113.24
Roraima	41.03	41.69	47.07	31.66	32.61	42.94	42.55	54.49	23.31	32.32	33.89	31.74	49.82	10.96	31.75
Santa Catarina	4603.41	3640.48	6060.43	6505.16	7300.24	4662.08	3977.06	5984.56	5698.92	5988.33	4757.92	4327.28	5941.42	4454.28	5137.64
São Paulo	41390.85	27064.96	30532.63	40081.26	45227.88	38002.86	28002.72	29791.31	33216.99	35163.06	32970.11	27839.26	28339.81	26339.96	29146.58
Sergipe	74.54	113.39	114.19	142.34	68.22	61.48	96.37	110.25	39.09	61.31	52.02	70.72	99.32	16.12	67.7
Tocantins	168.36	226.38	199.4	56.62	129.67	134.43	180.71	236.55	37.44	114.94	93.28	118.04	235.88	26.85	118.99

Table A: RMSE obtained for each state and nowcasting approach for previous one to three weeks respectively. Red and blue represent the best and the second best performances respectively (the lowest and the second lowest error).

	Past one week					Past two weeks					Past three weeks				
	DCGT	DC	GT	InfoDengue	Naive	DCGT	DC	GT	InfoDengue	Naive	DCGT	DC	GT	InfoDengue	Naive
Acre	0.48	0.49	0.49	0.6	0.5	0.36	0.36	0.47	0.18	0.34	0.24	0.24	0.47	0.13	0.27
Alagoas	0.49	0.71	0.36	0.69	0.72	0.38	0.64	0.37	0.28	0.56	0.37	0.46	0.38	0.19	0.47
Amapá	2.53	2.44	1.11	3	47.36	2.59	2.55	1.55	2.47	9.68	3.5	3.47	1.01	2.01	4.73
Amazonas	0.27	0.32	0.87	0.33	0.44	0.23	0.25	0.41	0.2	0.28	0.19	0.17	6.67	0.12	0.19
Bahia	0.36	0.49	0.52	0.38	0.5	0.27	0.35	0.52	0.21	0.3	0.22	0.21	0.58	0.14	0.24
Ceará	0.35	0.41	0.49	0.56	0.57	0.28	0.34	0.48	0.18	0.3	0.22	0.23	0.48	0.1	0.25
Distrito Federal	0.46	0.4	0.37	0.19	0.28	0.38	0.32	0.34	0.13	0.25	0.25	0.21	0.34	0.1	0.2
Espírito Santo	-	-	-	-	-	-	-	-	-	-	-	-	-	-	-
Goiás	0.46	0.52	0.29	0.27	0.53	0.41	0.48	0.32	0.22	0.31	0.38	0.44	0.28	0.16	0.22
Maranhão	0.38	0.64	6.57	1.22	1.54	0.32	0.47	0.59	0.77	0.48	0.26	0.31	0.29	0.32	0.34
Mato Grosso	0.31	0.52	0.22	0.23	0.52	0.26	0.35	0.19	0.19	0.35	0.25	0.26	0.19	0.16	0.26
Mato Grosso do Sul	0.34	0.32	0.29	0.69	0.71	0.26	0.26	0.31	0.37	0.46	0.28	0.33	0.34	0.29	0.32
Minas Gerais	1.68	1.96	0.3	2.64	2.55	1.38	1.54	0.29	1.55	1.51	1.18	1.24	0.3	1.07	1.06
Pará	0.88	1.42	0.36	0.54	1.45	0.69	0.88	0.33	0.42	0.86	0.48	0.52	0.3	0.35	0.48
Paraíba	0.43	0.84	0.22	0.15	0.32	0.32	0.57	0.29	0.1	0.33	0.22	0.29	0.22	0.09	0.33
Paraná	0.26	0.27	0.2	0.3	0.28	0.25	0.24	0.21	0.11	0.2	0.19	0.17	0.2	0.09	0.18
Pernambuco	0.31	0.6	0.24	0.35	0.59	0.29	0.5	0.23	0.16	0.43	0.26	0.33	0.23	0.13	0.36
Piauí	0.48	0.56	0.5	0.35	0.82	0.4	0.48	0.36	0.27	0.59	0.36	0.32	0.42	0.19	0.38
Rio de Janeiro	0.33	0.36	0.28	0.22	0.59	0.29	0.3	0.29	0.18	0.37	0.28	0.29	0.26	0.18	0.25
Rio Grande do Norte	0.27	0.6	0.24	0.28	0.27	0.24	0.49	0.23	0.09	0.27	0.2	0.26	0.26	0.08	0.29
Rio Grande do Sul	0.42	0.39	0.43	0.46	0.42	0.33	0.33	0.41	0.22	0.34	0.26	0.25	0.45	0.17	0.29
Rondônia	0.49	0.58	6.14	1.32	2	0.39	0.44	0.46	0.76	1.15	0.34	0.36	0.75	0.43	0.46
Roraima	0.44	0.44	0.74	0.43	0.51	0.44	0.44	1.07	0.23	0.42	0.38	0.35	0.91	0.14	0.37
Santa Catarina	0.29	0.29	0.46	0.4	0.39	0.25	0.25	0.43	0.23	0.3	0.21	0.2	0.43	0.17	0.26
São Paulo	0.45	0.35	0.26	0.38	0.47	0.36	0.3	0.26	0.26	0.33	0.28	0.24	0.23	0.18	0.24
Sergipe	0.26	0.44	0.36	0.38	0.38	0.26	0.37	0.33	0.17	0.21	0.22	0.23	0.33	0.06	0.23
Tocantins	0.33	0.37	0.42	0.15	0.27	0.26	0.29	0.56	0.07	0.23	0.19	0.21	0.48	0.05	0.2

Table B: RMSPE obtained for each state and nowcasting approach for previous one to three weeks respectively. Red and blue represent the best and the second best performances respectively (the lowest and the second lowest error).

	Past one week			Past two weeks			Past three weeks		
	DCGT	DC	GT	DCGT	DC	GT	DCGT	DC	GT
Acre	0.96	0.98	0.89	0.98	0.98	0.91	0.98	0.98	0.93
Alagoas	0.89	0.82	1	0.82	0.82	0.98	0.8	0.8	1
Amapá	0.78	0.78	0.82	0.56	0.58	0.8	0.4	0.38	0.8
Amazonas	1	0.96	1	1	1	1	1	1	1
Bahia	0.89	0.87	0.89	0.87	0.89	0.82	0.91	0.91	0.78
Ceará	0.91	0.91	0.93	0.91	0.89	0.96	0.89	0.93	0.96
Distrito Federal	0.87	0.89	0.93	0.89	0.93	0.91	0.91	0.98	0.93
Espírito Santo	-	-	-	-	-	-	-	-	-
Goiás	0.97	0.97	0.78	0.97	0.97	0.78	0.94	1	0.8
Maranhão	0.96	0.89	0.87	0.98	0.89	0.91	0.93	0.91	0.91
Mato Grosso	0.93	0.87	1	0.91	0.84	0.98	0.91	0.89	1
Mato Grosso do Sul	1	1	1	1	1	1	0.96	0.96	0.98
Minas Gerais	0.76	0.78	0.98	0.71	0.71	0.98	0.44	0.47	0.98
Pará	0.87	0.93	0.89	0.89	0.96	0.89	0.82	0.84	0.93
Paraíba	0.93	0.91	0.98	0.93	0.96	0.96	0.89	0.87	0.98
Paraná	0.98	0.96	0.98	1	1	0.98	0.89	0.96	0.98
Pernambuco	0.82	0.93	1	0.84	0.87	0.98	0.76	0.78	1
Piauí	0.98	0.98	0.96	0.98	0.96	0.96	0.87	0.96	0.98
Rio de Janeiro	0.98	0.96	1	0.98	0.96	1	0.89	0.87	1
Rio Grande do Norte	0.93	0.96	1	0.96	0.93	1	0.96	0.93	1
Rio Grande do Sul	0.91	0.98	0.84	0.93	0.98	0.84	0.87	1	0.84
Rondônia	1	1	0.98	0.98	0.98	1	1	1	0.98
Roraima	0.96	0.96	0.93	0.89	0.89	0.93	0.89	0.89	0.91
Santa Catarina	0.98	0.98	0.78	1	1	0.82	0.93	0.98	0.78
São Paulo	0.71	0.87	0.96	0.71	0.8	0.93	0.67	0.71	0.96
Sergipe	0.96	0.93	0.98	0.96	0.91	1	0.91	0.91	1
Tocantins	1	1	0.96	1	1	0.93	1	1	0.98

Table C: 95% coverage probabilities obtained for each state and nowcasting approach for previous one to three weeks respectively. Red represent models closest to nominal coverage.

	Past one week			Past two weeks			Past three weeks		
	DCGT	DC	GT	DCGT	DC	GT	DCGT	DC	GT
Acre	0.58	0.56	0.58	0.67	0.64	0.42	0.71	0.71	0.42
Alagoas	0.4	0.49	0.64	0.33	0.33	0.6	0.31	0.33	0.64
Amapá	0.24	0.31	0.36	0.2	0.16	0.44	0.04	0.04	0.42
Amazonas	0.58	0.69	0.51	0.62	0.67	0.6	0.62	0.67	0.51
Bahia	0.69	0.64	0.4	0.73	0.73	0.49	0.58	0.6	0.42
Ceará	0.76	0.71	0.49	0.73	0.76	0.44	0.67	0.69	0.49
Distrito Federal	0.49	0.49	0.29	0.44	0.42	0.31	0.42	0.33	0.27
Espírito Santo	-	-	-	-	-	-	-	-	-
Goiás	0.73	0.67	0.38	0.73	0.75	0.42	0.74	0.73	0.44
Maranhão	0.64	0.6	0.56	0.64	0.67	0.53	0.56	0.53	0.58
Mato Grosso	0.6	0.56	0.64	0.64	0.58	0.62	0.58	0.51	0.67
Mato Grosso do Sul	0.8	0.8	0.51	0.8	0.8	0.49	0.62	0.6	0.44
Minas Gerais	0.33	0.47	0.49	0.22	0.29	0.49	0.04	0.07	0.47
Pará	0.42	0.6	0.29	0.36	0.49	0.36	0.2	0.31	0.44
Paraíba	0.78	0.76	0.6	0.71	0.76	0.53	0.53	0.64	0.62
Paraná	0.73	0.76	0.62	0.62	0.71	0.56	0.64	0.6	0.53
Pernambuco	0.69	0.62	0.71	0.56	0.56	0.64	0.38	0.31	0.71
Piauí	0.64	0.76	0.51	0.64	0.69	0.51	0.6	0.62	0.44
Rio de Janeiro	0.51	0.53	0.53	0.33	0.4	0.47	0.18	0.24	0.58
Rio Grande do Norte	0.69	0.64	0.62	0.62	0.69	0.58	0.6	0.73	0.62
Rio Grande do Sul	0.6	0.71	0.49	0.56	0.71	0.56	0.47	0.53	0.49
Rondônia	0.8	0.8	0.4	0.84	0.87	0.4	0.73	0.76	0.51
Roraima	0.6	0.6	0.56	0.64	0.62	0.36	0.49	0.47	0.4
Santa Catarina	0.67	0.71	0.4	0.56	0.58	0.36	0.42	0.51	0.31
São Paulo	0.44	0.53	0.62	0.42	0.51	0.53	0.44	0.4	0.56
Sergipe	0.67	0.64	0.51	0.64	0.71	0.53	0.62	0.73	0.6
Tocantins	0.73	0.58	0.51	0.71	0.67	0.47	0.71	0.71	0.38

Table D: 50% coverage probabilities obtained for each state and nowcasting approach for previous one to three weeks respectively. Red represent models closest to nominal coverage.

	Past one week			Past two weeks			Past three weeks		
	DCGT	DC	GT	DCGT	DC	GT	DCGT	DC	GT
Acre	6.53	6.55	6.46	6.21	6.23	6.38	5.8	5.8	6.36
Alagoas	6.89	7.52	6.59	6.71	7.86	6.73	7.26	7.81	6.78
Amapá	9.67	9.51	7.23	11.54	11.38	8.45	23.4	23.04	7.25
Amazonas	6.07	6.49	6.29	5.98	6.24	6.29	5.82	5.9	6.34
Bahia	8.61	9.18	10.35	8.23	8.86	9.95	7.96	8.07	8.92
Ceará	7.01	7.16	8.36	6.83	7.02	8.46	6.62	6.73	8.39
Distrito Federal	7.64	7.54	7.91	7.55	7.34	7.85	7.14	6.97	7.91
Espírito Santo	-	-	-	-	-	-	-	-	-
Goiás	8.28	8.43	8.4	8.07	8.17	8.5	7.85	7.82	8.5
Maranhão	5.55	6.71	6.07	5.4	6.19	5.71	5.11	5.44	5.62
Mato Grosso	7.49	8.22	7.07	7.23	7.67	7.05	7.04	7.21	7.03
Mato Grosso do Sul	7.16	7.3	6.89	6.93	7.05	6.98	6.63	6.72	7.06
Minas Gerais	15.82	17.35	9.55	17.2	18.59	9.69	25.5	26.52	9.64
Pará	8.8	9.44	6.86	8.69	8.88	6.66	8.62	8.41	6.71
Paraíba	7.7	9.07	6.22	7.15	8.32	6.54	6.45	6.96	6.47
Paraná	9.22	9.33	8.78	9	9.05	8.91	8.87	8.69	8.93
Pernambuco	8.03	8.55	7.21	8.12	8.63	7.23	8.56	8.67	7.27
Piauí	6	6.05	5.85	5.8	5.88	5.78	5.75	5.63	5.87
Rio de Janeiro	8.01	8.15	8.15	7.88	7.95	8.21	8.15	8.14	8.18
Rio Grande do Norte	6.38	7.12	6.15	6.22	6.9	6.26	5.91	6.23	6.34
Rio Grande do Sul	8.12	8.05	8.51	7.85	7.74	8.43	7.55	7.31	8.34
Rondônia	5.82	6.13	6.19	5.59	5.78	5.99	5.15	5.23	6.15
Roraima	5.15	5.16	5.37	5.29	5.3	5.59	5.1	4.94	5.42
Santa Catarina	8.84	8.88	9.73	8.6	8.6	9.65	8.39	8.31	9.77
São Paulo	12.84	11.11	10.51	13.11	11.32	10.54	14.63	12.54	10.5
Sergipe	5.62	6.26	5.95	5.43	6.19	5.91	5.29	5.71	5.93
Tocantins	6.3	6.41	6.59	6.07	6.16	6.78	5.73	5.8	6.73

Table E: Logscore obtained for each state and nowcasting approach for previous one to three weeks respectively. Red and blue represent the best and the second best performances respectively (the lowest and the second lowest logscore).
